# Atrial Fibrillation in Asia and Globally: “Easy as ABC,” “It’s an SOS,” or “Handle With CARE”

**DOI:** 10.1016/j.jacasi.2024.11.013

**Published:** 2025-01-21

**Authors:** Gregory Y.H. Lip

**Keywords:** ABC pathway, Asia, atrial fibrillation, management

**Figure:**
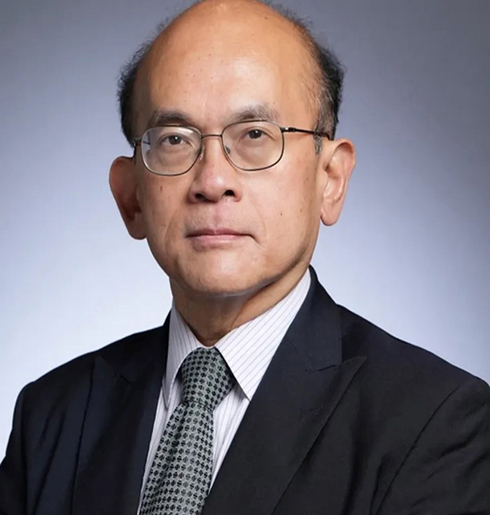

Atrial fibrillation (AF) is the most common cardiac rhythm disorder globally, and clearly remains a health care problem in Asia, with its increasingly aged population.^1^ AF is associated with a high mortality and morbidity, commonly from stroke, heart failure, and hospitalizations^2^—but the tsunami of AF-related dementia and cognitive impairment is also increasingly recognized.^3^

Hence, it is so important to have contemporary data on the clinical epidemiology of AF from Asia, given the recognized racial differences in AF-related complications, such as stroke and bleeding.^4^^,^^5^ Numerous registries from Asia have been published in recent years that have provided valuable evidence on risk prediction, bleeding risks, and management (including data from the Asia-Pacific Heart Rhythm Society,^6^ the Indian KERALA-AF registry^7^ and the COOL-AF [COhort of antithrombotic use and Optimal INR Level in patients with non-valvular Atrial Fibrillation in Thailand] registry from Thailand^8^^,^^9^) that have informed management guidelines from Asia.^10^, ^11^, ^12^

In this issue of *JACC: Asia*, Krittayaphong et al^13^ describe plans for the COOL-AF Phase 2 study, which is a prospective observational multicenter study of patients with known or newly diagnosed nonvalvular AF in Thailand, aiming to establish a prospective cohort of 3,667 AF patients from 33 centers who will be followed up every 6 months for up to 3 years. The careful follow-up with prospective documentation of comorbidities and outcomes is important, given that risk is not static, but dynamic, and builds on the similarly successful prior outputs from COOL-AF showing the changes in multimorbidity and compliance with the evidence-based ABC (Atrial fibrillation Better Care) pathway over time, and how they affect outcomes.^14^^,^^15^ The ABC pathway is supported by trial and real-world evidence (even from Asia),^6^^,^^16^^,^^17^ and variants of the “ABC pathway” acronym have been used in the recent U.S. guidelines (ie, SOS [Stroke, Other Comorbidities, Rate or Rhythm control])^18^ and 2024 European guidelines^19^ (as CARE [Comorbidities, Avoid stroke, Rate or rhythm control, Evaluation])^20^ (Figure 1).

**Figure 1:**
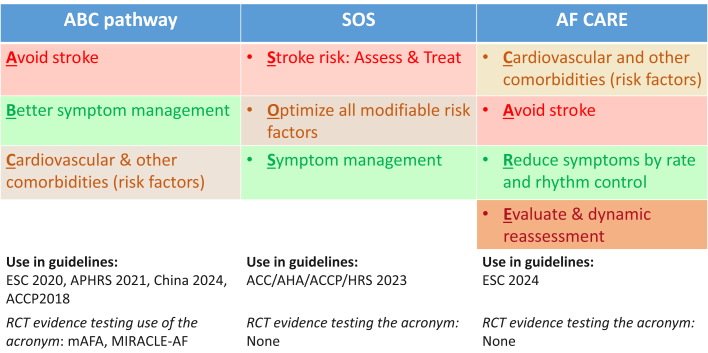
The ABC, “SOS,” and AF-CARE Pathways From the Various Published Guidelines on AF Management Adapted with permission from Potpara et al.^20^ ACC = American College of Cardiology; ACCP = American College of Clinical Pharmacy; AHA = American Heart Association; APHRS = Asia-Pacific Heart Rhythm Society; ESC = European Society of Cardiology; HRS = Heart Rhythm Society; mAFA = mobile AF App trial; MIRACLE-AF = a novel Model of IntegRAted Care of oLdEr patients with Atrial Fibrillation in rural China trial; RCT = randomized controlled trial.

Nevertheless, it is the patients with known AF who are included in registries, but many AF patients are asymptomatic and undetected; therefore, efforts have been directed toward screening for this condition. Following a national AF screening project in Taiwan,^21^ the paper in this issue of *JACC: Asia* by Fu et al^22^ evaluated the cost-effectiveness of this population screening program for AF in the elderly. Using a Markov decision-analytic model, both one-time and annual population screening for AF in individuals aged 65 to 80 were cost-effective. This is consistent with other analyses highlighting the health care cost impact of targeted vs systematic AF screening.^23^

Following the diagnosis of AF, the appropriate evaluation and characterization of AF patients is needed, and the 4S (Stroke assessment, Symptoms, Severity of burden and Substrate) scheme is a simple approach to do this.^24^ This has been applied to the Asia-Pacific Heart Rhythm Society registry population, showing how categorization according to the 4S-AF scheme was related to the adverse outcomes in Asian AF patients, and appropriate treatments based on the 4S-AF scheme translated to better clinical outcomes.^25^

Next requires the management of AF, which has evolved toward a holistic or integrated care approach, with the ABC pathway.^11^ Avoiding stroke is a key pillar of AF management whereby the default should be to offer stroke prevention to all patients with AF, unless they are low risk. In many Asian countries, vitamin K antagonists such as warfarin remain the first choice of oral anticoagulant (OAC), but the challenge is to maintain good quality anticoagulation control, as reflected by the time in therapeutic range (TTR). The SAMe-TT_2_2R_2_ score was a simple clinical score first proposed to help identify those patients likely to do poorly on warfarin (ie, SAMe-TT_2_2R_2_ score [SAMe-TT2R2 score, awarding 1 point for each of the following: sex (female); age <60 years; medical history (≥3 comorbidities: hypertension, diabetes, coronary heart disease/myocardial infarction, peripheral artery disease, heart failure, prior stroke, lung disease, liver disease or renal failure); treatments (such as amiodarone); smoking status (double); and race (non-Caucasian, double)] >2) who could be identified for more careful review, follow-up, and education.^26^

Also in this issue of *JACC: Asia*, Phrommintikul et al^27^ report the primary results of the TREATS-AF (A prospective randomised trial examining the impact of an intensive educational intervention versus usual care on anticoagulation therapy control intervention based on SAMe-TT2R2 score-guided strategy in anticoagulant-naive Thai patients with atrial fibrillation) trial which investigated the impact of educational-behavioral intervention on anticoagulation control using SAMe-TT_2_2R_2_ score-guided strategy in AF. In this setting with overall poor anticoagulation control, TREATS-AF showed that a SAMe-TT_2_R_2_ score-guided strategy did not significantly improve outcomes (TTR at 12 months) over 12 months, compared with usual care. This suggests that in Asian patients, we may need more than educational-behavioral intervention to improve TTR, and given the increasing availability of generic direct OACs, this may ultimately be the preferred option for an OAC strategy.

Nevertheless, not all patients would be able to take long-term OAC therapy, and interventional procedures such as left atrial appendage closure (LAAC) are increasingly used globally, especially in high bleeding-risk patients such as those with end-stage renal failure. In this issue of *JACC: Asia*, Tanaka et al^28^ report the OCEAN-LAAC (Outcomes of Left Atrial Appendage Closure in Hemodialysis Patients With Atrial Fibrillation) registry, which aimed to report the benefits and harms of LAAC for hemodialysis (HD) patients. Reassuringly, this observational cohort shows that LAAC is safe for HD patients and achieves results comparable to those in non-HD patients.

The ‘B’ in the ABC pathway refers to patient-centered, symptom-directed decisions on rate or rhythm control. For the latter, numerous advances in rhythm control have been described, and for AF catheter ablation, there has been great interest in pulse field ablation (PFA), which offers shorter procedure times and comparable outcomes to conventional pulmonary vein isolation using radiofrequency or cryoablation. In this issue of *JACC: Asia*, Li et al^29^ reported a meta-analysis aimed to investigate the effectiveness and safety of PFA in treating AF patients and compared its outcomes with conventional thermal ablation. They demonstrated that PFA showed noninferiority to thermal ablation in acute pulmonary vein isolation and superiority in first-pass isolation, atrial arrhythmia recurrence, phrenic nerve paralysis or injury, and procedure time. However, PFA treatment was associated with a higher risk of cardiac perforation or tamponade.

The ‘C’ of the ABC pathway refers of comorbidity and risk factor management, including attention to psychological morbidity and lifestyle factors. For the latter, obesity is commonly associated with incident AF and AF-related complications. In this issue of *JACC: Asia*, Rhee et al^30^ investigated how to best evaluate the impact of obesity, because the traditional focus on body mass index (BMI) has many limitations. They show the impact of the cumulative burden of BMI and waist circumference (WC) and their combination on the incident AF risk. In the Korean nationwide cohort study, the cumulative burden of WC was a better indicator of AF risk than either a single BMI measurement or the overall BMI burden. Indeed, the cumulative burden of WC over 4 years was a more potent predictor of future AF risk than either a single BMI measurement or the overall BMI burden.

This theme issue of *JACC: Asia* clearly shows a diverse selection of the studies related to AF screening, and the components of the holistic or integrated care management of this common condition. Clearly, there remains so much more to do and learn to improve the care and management of our patients with AF.

## Funding Support and Author Disclosures

Dr Lip is a National Institute for Health and Care Research (NIHR) Senior Investigator; a Consultant and speaker for BMS/Pfizer, Boehringer Ingelheim, Daiichi-Sankyo, and Anthos (no fees are received personally); is co-PI of the AFFIRMO project on multimorbidity in AF (grant agreement No. 899871), TARGET project on digital twins for personalized management of atrial fibrillation and stroke (grant agreement No. 101136244), and ARISTOTELES project on artificial intelligence for management of chronic long term conditions (grant agreement No. 101080189), which are all funded by the EUs Horizon Europe Research and Innovation programme.
